# Contextually adapted functional and dysfunctional presenteeism during protracted conflict

**DOI:** 10.3389/fpsyg.2026.1903610

**Published:** 2026-09-16

**Authors:** Galia Sheffer-Hilel, Einav Srulovici, Ilana Choen, Anat Drach-Zahavy

**Affiliations:** 1 Department of Nutrition Sciences, Faculty of Sciences, Tel-Hai Academic College, Kiryat Shmona, Israel; 2 The Cheryl Spencer Department of Nursing, Faculty of Social Welfare and Health Sciences at Haifa University, Haifa, Israel

**Keywords:** healthcare-professionals, organizational trust, presenteeism, resilience, strain

## Abstract

**Background:**

Healthcare professionals working during protracted conflict are exposed to persistent stressors that challenge both psychological well-being and occupational functioning. Understanding the mechanisms that support adaptive functioning under prolonged adversity is essential for maintaining workforce capacity and continuity of care. Drawing on Conservation of Resources (COR) theory, this study examined how stress, resilience, organizational trust, and strain symptoms relate to functional and dysfunctional presenteeism.

**Objective:**

To examine whether strain symptoms mediate the relationships between perceived stress with dysfunctional presenteeism and the relationships of personal resilience and organizational trust with functional presenteeism among healthcare professionals during protracted conflict.

**Methods:**

A cross-sectional study was conducted among 227 healthcare professionals. Participants completed self-report measures of perceived stress, personal resilience, organizational trust, strain symptoms, and work functioning. The Stanford Presenteeism Scale (SPS-6) was contextually adapted to assess work functioning during protracted armed conflict. Path analysis with bootstrapping (5,000 resamples) was used to test the hypothesized relationships.

**Results:**

Participants reported relatively high functional presenteeism (*M* = 3.85, SD = 0.77) and lower dysfunctional presenteeism (*M* = 2.79, SD = 0.98). The final model demonstrated acceptable-to-good fit [χ^2^(9) = 12.48, *p* = 0.188, CFI = 0.978, TLI = 0.949, RMSEA = 0.042]. Resilience and organizational trust were associated with higher functional presenteeism through lower strain symptoms. In contrast, perceived stress showed a significant direct association with dysfunctional presenteeism (β = 0.17, *p* = 0.005), while its indirect effect through strain symptoms was not significant.

**Conclusion:**

Findings provide partial support for the proposed model and suggest distinct patterns of functional and dysfunctional presenteeism during protracted conflict, as captured by the contextually adapted framework. Perceived stress was primarily associated with maladaptive work functioning, whereas personal resilience and organizational trust were associated with adaptive functioning through lower strain symptoms. Strengthening individual and organizational resources may help nurses and dietitians, and potentially other healthcare professionals, maintain effective functioning under prolonged adversity.

## Introduction

Presenteeism, namely the act of attending work while experiencing physical or mental health challenges, is a significant issue in healthcare, where its prevalence consistently exceeds that of other sectors (Antczak and Miszczyńska, 2024). High rates of presenteeism take a toll on both healthcare professionals’ well-being and patient care, leading to reduced performance, increased medical errors, and deteriorating health outcomes (Lui et al., 2018). Although previous research has identified key antecedents and consequences of presenteeism, much of the healthcare literature conceptualizes it as a uniform behavior. In fact, this approach overlooks the nuanced distinction between two distinct forms: dysfunctional presenteeism, which emerges from external attendance pressures and often leads to further physical and psychological deterioration; and functional presenteeism, which reflects adaptive strategies that allow professionals to maintain their work effectiveness despite minor health disruptions (Karanika-Murray and Biron, 2020; Patel et al., 2023).

More recently, the scope of presenteeism research has begun to extend beyond attendance during individual illness to examine work functioning under large-scale crises that generate substantial psychological strain. Studies conducted during the Coronavirus disease 2019 (COVID-19) pandemic (White-Means et al., 2022) and following earthquakes (Mete et al., 2024) suggest that healthcare professionals may continue working despite heightened anxiety, emotional burden, organizational disruption, and persistent uncertainty. These contexts share important features: both expose healthcare professionals to collective threat and sustained stress that may impair well-being and functioning even in the absence of acute physical illness. It is therefore important to broaden our understanding of the conditions under which functional and dysfunctional presenteeism emerge, particularly in contexts such as prolonged armed conflict, where chronic threat and uncertainty may affect employees’ physical and mental health and shape how they continue functioning at work.

This study addresses these gaps by developing and empirically testing a resilience-oriented model of work functioning during protracted conflict, grounded in Conservation of Resources (COR) theory (Hobfoll, 1989; Figure 1). The model applies a contextually adapted functional–dysfunctional presenteeism framework to examine continued work under the chronic psychological and situational strain of prolonged armed conflict. The model hypothesizes that strain symptoms, psychological, physiological, or behavioral manifestations of prolonged stress, and reduced well-being (Quick and Henderson, 2016), may mediate the association between perceived stress and dysfunctional presenteeism, as well as the associations of personal resilience and organizational trust with functional presenteeism. In line with COR theory, strain symptoms are conceptualized as a proximal response to threatened or depleted resources and as a potential mediating mechanism linking demands and resources with behavioral outcomes such as presenteeism. Within this framework, perceived stress is viewed as a workplace demand, whereas resilience and organizational trust function as key resources. Resilience represents a personal resource that reflects an individual’s capacity to adapt effectively to stress and adversity (Smith et al., 2008). Organizational trust, in turn, constitutes a social resource encompassing employees’ belief that the organization acts in their best interests, provides support, and maintains transparency (Yilmaz, 2019). In doing so, the study clarifies the dual structure of presenteeism and highlights the mechanisms through which individuals navigate resource loss and preservation in high-pressure clinical environments. By situating the model within a protracted conflict, the study pushes the boundaries of existing presenteeism research beyond acute crisis or stable periods. Its findings are expected to inform evidence-based interventions that promote functional coping, enhance psychological safety, and mitigate the risks associated with maladaptive presenteeism behaviors in healthcare systems under chronic strain.

**FIGURE 1:**
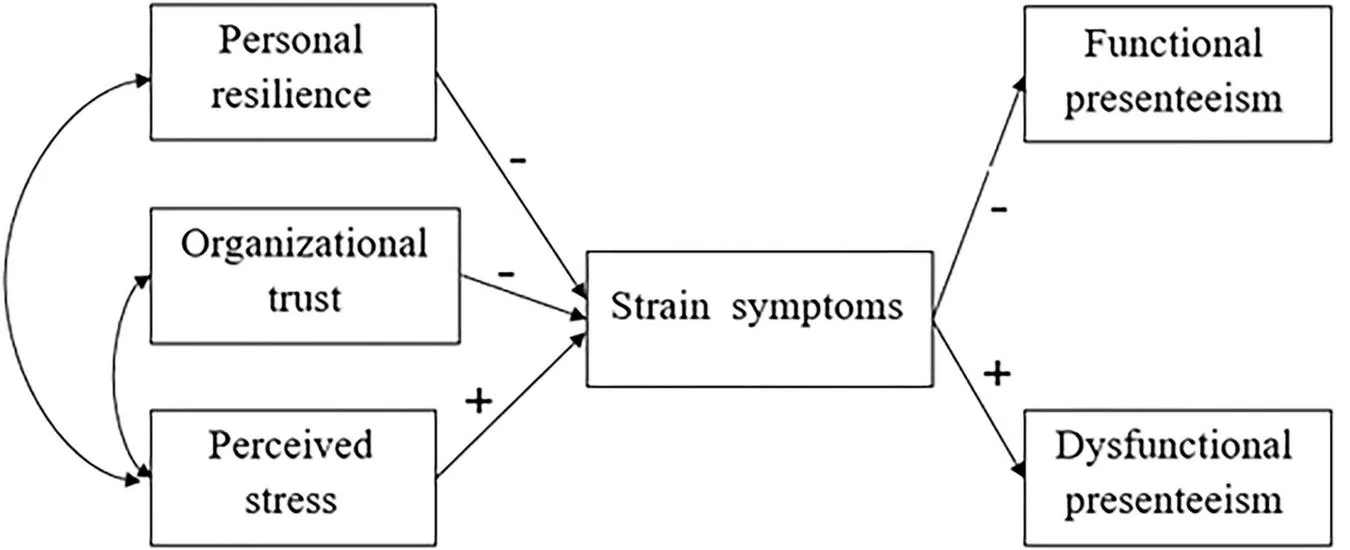
A resilience-oriented research model of presenteeism.

## Background

Presenteeism in healthcare refers to the behavior of healthcare professionals attending work despite experiencing poor physical or mental health, which leads to reduced performance and worsened health status (Lui et al., 2018). Evidence from a study conducted across European countries demonstrates that its prevalence in healthcare is significantly higher than in other occupational sectors (Antczak and Miszczyńska, 2024). A systematic review found that 86% of general practitioners and over 50% of hospital physicians in the United Kingdom (UK) reported engaging in presenteeism, compared to just 32% of office workers (Lui et al., 2018). Among medical residents, 51% reported working with flu-like symptoms at least once in the previous year, and 9.1% of physicians and nurses with respiratory symptoms tested positive for influenza while continuing to work in Brazil (Homrich et al., 2020). Gender differences have been observed, with women more likely to engage in presenteeism due to concern for others and workload, whereas men more often cite ability and financial reasons as motivating factors (Antczak and Miszczyńska, 2024). While most research has focused on physicians and nurses, presenteeism has also been reported among other healthcare professionals, including physical therapists and occupational therapists (Torp and Bergheim, 2023). However, evidence regarding other allied health professionals, particularly dietitians, remains limited.

The causes of presenteeism in healthcare are complex and multifaceted. High job demands, such as heavy workload, time pressure, and staff shortages, are consistent drivers of presenteeism (Navarro et al., 2018). In addition, professional norms and workplace culture play a key role, as healthcare workers often feel obligated to continue working while ill due to the perceived impact on patient care and team performance (Lui et al., 2018). Emotional factors, such as fear of professional judgment and concerns about burdening colleagues, further reinforce presenteeism behavior. Lastly, organizational factors, including lack of backup staffing and pressure from supervisors, contribute to this trend (Lui et al., 2018). Although these factors may compel healthcare workers to attend work while ill, they do so at considerable cost to both individual well-being and the safety and functioning of healthcare systems.

The cost of presenteeism to individual healthcare workers’ health is significant, encompassing increased anxiety (17.5%), depression (22.7%), and sleep problems (26%) (Homrich et al., 2020). Presenteeism also leads to reduced concentration and decision-making ability, resulting in adverse outcomes for healthcare systems, such as higher rates of medical errors, patient falls, and disease transmission. The most recent estimates available of productivity loss due to presenteeism range from $2,000 to over $15,000 per healthcare employee annually (Lui et al., 2018).

Despite growing interest in presenteeism, research in healthcare remains limited by persistent conceptual ambiguity, partially because presenteeism has been traditionally treated as the single behavior of attending work while unwell. This definition conflates two distinct, though interrelated, dimensions: the presence of a physical or mental health condition (i.e., health) and the decision to attend work (i.e., attendance) (Homrich et al., 2020; Lui et al., 2018; McGregor and Caputi, 2022). Yet, conceptually, presenteeism may be better understood as arising from the combination of these two dimensions rather than from either dimension alone. In other words, it involves both a health condition and attendance at work despite that condition. For example, two healthcare professionals with comparable health conditions may make different attendance decisions, whereas two professionals who both attend work may differ substantially in the severity of their health condition.

To address this gap, recent scholars outside the healthcare sector have advocated a dual conceptualization of presenteeism, viewing it as the dynamic interplay between health status and attendance behavior, each shaped by personal, organizational, and social factors (Lohaus et al., 2021; Patel et al., 2023). Within this perspective, dysfunctional presenteeism reflects externally driven attendance that impairs functioning and health, whereas functional presenteeism reflects attendance despite manageable health challenges while maintaining effective functioning and deriving psychological or professional benefits, such as preserving professional identity, maintaining emotional stability, or supporting colleagues (Karanika-Murray and Biron, 2020). This refined conceptualization underscores the need for research that distinguishes not only who attends work despite health challenges, but also why they do so.

Furthermore, despite recognizing both physical and mental health conditions, presenteeism research has focused primarily on illness-related attendance. This broader perspective is consistent with research showing that emotional distress, including anxiety, depression, and traumatic stress responses, has indeed been associated with presenteeism (Mosteiro-Diaz et al., 2023). During the COVID-19 pandemic, crisis-specific stressors–such as inadequate protective equipment, heightened professional responsibility, and severe staff shortages–appeared to intensify this phenomenon. White-Means et al. (2022) reported presenteeism rates of 77% among nurses and respiratory therapists, substantially higher than the pre-pandemic rates of approximately 40%–50% reported in earlier studies (Baldonedo-Mosteiro et al., 2020; Warren et al., 2011). The consequences were considerable: 32% of healthcare workers reported productivity losses of at least 40%, while depression and anxiety were reported by 68% and 67% of participants, respectively approximately twice and four times their pre-pandemic levels. Traumatic stress responses, including nightmares and intrusive thoughts, further undermined work performance (White-Means et al., 2022).

A comparable pattern was observed following the 2023 earthquakes in Türkiye. Mete et al. (2024) examined presenteeism among physicians working in an affected region and described several crisis-related conditions that could impair work functioning, including post-earthquake psychological stress, disrupted work routines, increased workload, workplace damage, safety concerns, and the dual burden of being both disaster victims and frontline responders. Their findings further showed that coping strategies were associated with presenteeism: positive reappraisal was related to lower presenteeism, whereas greater reliance on social support was associated with higher levels. Together, the COVID-19 and earthquake studies suggest that, in large-scale crises, presenteeism may reflect continued work under substantial psychological, organizational, and situational strain rather than only attendance despite a conventional physical illness.

Taking together, these conceptual and contextual developments suggest that presenteeism is best understood as a context-dependent process that may take either a functional or dysfunctional form. Yet little is known about the mechanisms that determine which form emerges when healthcare professionals continue working under prolonged psychological and situational strain, such as during protracted armed conflict. Although such professionals may not be physically ill, prolonged exposure to war has been associated with anxiety, traumatic stress, sleep disturbances, and other indicators of impaired mental health (Marey-Sarwan et al., 2022; Sydorenko et al., 2025), thereby creating health-related conditions under which presenteeism may occur. Addressing this gap requires a theoretically grounded model explaining how perceived stress, personal resilience, organizational trust, and strain symptoms are associated with functional and dysfunctional presenteeism under prolonged adversity.

### A resilience-oriented research model for functional and dysfunctional presenteeism

The present study is based on the COR Theory (Hobfoll, 1989), which posits that individuals strive to acquire, maintain, and protect valuable resources to manage stress and avoid further resource loss. Based on this framework, we propose a resilience-oriented model in which strain symptoms (reflecting impaired well-being) mediate the relationships between perceived stress, personal resilience, organizational trust, and two distinct forms of presenteeism: functional and dysfunctional (Figure 1). According to COR, stress arises when individuals perceive a threat to valued resources, such as time, energy, or emotional stability, or experience actual resource loss (Hobfoll, 1989). This is particularly salient in healthcare, where heavy workloads, staff shortages, and high-stakes performance demands continuously deplete resources (Li and Hasson, 2020). During protracted conflict, these demands are further intensified as healthcare professionals work under unpredictable conditions, often without adequate guidelines or protective equipment (Hoernke et al., 2021). They must improvise care, cope with moral distress while treating severely injured patients, and manage concerns for their own safety and that of their families (Marey-Sarwan et al., 2022). Together, these demands increase strain (Sydorenko et al., 2025), thereby elevating the risk of strain symptoms (McCann et al., 2013).

According to COR, individuals facing demanding conditions rely on available resources to prevent resource loss and maintain functioning (Hobfoll, 1989). Although resources may be personal, organizational, material, or energetic, the present study focuses on two key protective resources that are particularly relevant in high-stress healthcare settings: personal resilience and organizational trust. These represent an internal psychological capacity and an organizational resource that together shape how professionals respond to adversity, manage strain, and ultimately enact functional or dysfunctional presenteeism (Hobfoll, 1989).

Personal resilience is a relatively stable personal trait that enables adaptation under pressure and sustained performance, allowing individuals to recover and thrive despite adversity (Hu et al., 2015; Li and Hasson, 2020; Ungar, 2011). Trust in the organization involves confidence in the knowledge that management will provide necessary support, ensure safety protocols, and communicate transparently in rapidly changing situations (Drach-Zahavy et al., 2022). In protracted conflict, when standard procedures may be insufficient or absent, and vital materials such as protective equipment may be lacking (Quinn et al., 2024), both resilience and trust serve as anchors. Namely, they enable professionals to improvise, sustain commitment, and withstand emotional fatigue even while working under acute threat. The perception that one’s organization is supportive can buffer strain, enhance morale, and reduce the intensity of strain symptoms (Luhmann, 2000).

The model further proposes that strain symptoms may shape how presenteeism is enacted. When strain symptoms are higher, individuals are more likely to engage in dysfunctional presenteeism, a maladaptive form of attendance driven by fear of resource loss (e.g., job security, professional reputation) (Karanika-Murray and Biron, 2020; Patel et al., 2023), which ultimately contributes to health deterioration and reduced performance (Vandenbroeck et al., 2017). Conversely, when strain symptoms are low, professionals who continue working under adversity may be better able to maintain concentration, energy, and task completion, thereby displaying a more functional pattern of presenteeism.

In summary, the model advances a contextual, mechanism-based understanding of presenteeism, consistent with the COR theory. It conceptualizes presenteeism not as a singular or inherently negative behavior, but as a context-sensitive coping strategy whose functional or dysfunctional form may be shaped by the dynamic interplay of perceived stress, available resources, and underlying psychological functioning. In applying this model during a period of wartime, the study contributes to refining presenteeism theory and offers a framework for targeted, resilience-oriented intervention.

### The study

Aim. To examine two complementary mechanisms of presenteeism among healthcare professionals during a protracted conflict (1) strain symptoms mediating the link between perceived stress and dysfunctional presenteeism, and (2) strain symptoms mediating the link between personal resilience and organizational trust and functional presenteeism.

### Hypotheses

Based on this aim, the study proposes the following hypotheses:

**Hypothesis 1**: Strain symptoms will mediate the relationship between perceived stress and dysfunctional presenteeism, such that greater perceived stress will be associated with more strain symptoms, which, in turn, will be associated with greater dysfunctional presenteeism.

**Hypothesis 2**: Strain symptoms will mediate the relationship between personal resilience and functional presenteeism, such that personal resilience will be associated with fewer strain symptoms, which, in turn, will be associated with greater functional presenteeism.

**Hypothesis 3**: Strain symptoms will mediate the relationship between organizational trust and functional presenteeism, such that organizational trust will be associated with fewer strain symptoms, which, in turn, will be associated with greater functional presenteeism.

## Materials and methods

### Design

A cross-sectional study with a hypothesis-driven mediation analysis was conducted between December 2023 and March 2024.

### Participants

A convenience sample of 227 participants (119 nurses and 108 dietitians) were recruited via a snowball sampling method, which allowed access to a wider and potentially hard-to-reach population through existing professional or social networks.

Although nurses and dietitians differ in their professional responsibilities, patterns of patient contact, organizational roles, and potentially in conflict-related exposure and pressures shaping presenteeism decisions, the present study focused on psychological and organizational mechanisms of presenteeism that were expected to operate across clinical healthcare professions rather than on profession-specific effects. Both groups continued to provide direct patient care throughout the protracted conflict and were exposed to sustained organizational and emotional demands relevant to the proposed model. They were therefore analyzed as a pooled sample of clinical healthcare professionals, with professional group examined separately in a sensitivity analysis to assess whether professional affiliation materially affected the observed structural associations.

Participants were included if they had a minimum of 6 months in the workplace and 1 year in the profession. G*Power calculations indicated that, to achieve a small-medium effect size (f^2^ = 0.075) with a power of at least 0.80 and a significance level of 0.05, the required sample size should be at least 214 participants.

### Procedure

An electronic flyer was distributed via professional social media platforms and email lists, targeting dietitians and nurses across clinical settings. The flyer included information about the study’s purpose and a secure link to an online questionnaire hosted on Qualtrics. Upon entering the link, participants viewed a detailed informed consent form outlining the study’s aims, the voluntary nature of their participation, and data confidentiality. Only those who consented proceeded to the questionnaire. The questionnaire included only closed-ended, validated scales and took approximately 20 min to complete. Responses were anonymous and no identifiable information was collected. At the end of the survey, participants were invited to forward the survey link to eligible colleagues working in clinical nutrition or nursing roles. A total of 504 healthcare professionals accessed the survey (306 dietitians and 198 nurses). Of these, 227 participants completed all study measures (108 dietitians and 119 nurses), yielding an overall completion rate of 45.0%. As the invitation was disseminated through multiple open professional channels and snowball sampling, the number of individuals exposed to the invitation could not be determined, and a conventional response rate was therefore not applicable.

### Measures

The study measures were based on established instruments. The SPS-6 was contextually adapted for the present study, whereas the remaining measures were administered in their established forms. Cronbach’s α values based on the present sample are reported in Table 1.

**TABLE 1:** Means, standard deviations (SD), Cronbach’s α^a^, and the correlation matrix of the research variables.

Characteristics		*M*	*SD*	1	2	3	4	5	6	7
1	Functional presenteeism	3.85	0.77	(0.86)	(0.86)	(0.89)	(0.80)	(0.81)	(0.92)	–
2	Dysfunctional presenteeism	2.79	0.98	−0.27**						
3	Strain symptoms	2.37	0.66	−0.29**	0.49**					
4	Perceived stress	10.98	3.55	−0.06	0.27**	0.21**				
5	Personal resilience	3.18	0.43	0.26**	−0.27**	−0.37**	−0.16*			
6	Organizational trust	4.87	1.10	0.30**	−0.16*	−0.29**	0.09	−0.16*		
7	Professional tenure	14.15	12.90	0.03	−0.03	−0.09	−0.25	0.13	0.14	

*M*, mean; *SD*, standard deviation; *p < 0.05, **p < 0.01. ^a^Cronbach’s α values for all scales were calculated from the present study’s data and are reported on the diagonal.

Presenteeism was assessed using a contextually adapted version of the six-item Stanford Presenteeism Scale (SPS-6), originally developed to assess work functioning while experiencing a health problem (Koopman et al., 2002). References to illness or health problems were replaced with references to the period of protracted armed conflict, while the original item structure and response scale were retained. This adaptation was informed by evidence that prolonged exposure to armed conflict may generate sustained psychological strain and related health difficulties that affect occupational functioning. The adapted items were intended to capture relatively maintained versus impaired work functioning among healthcare professionals who continued working during prolonged adversity. Participants rated each item on a 5-point Likert scale (1 = strongly disagree to 5 = strongly agree).

Following the established two-dimensional structure of the SPS-6 and previous psychometric evidence supporting separate dimensions (Bezzina et al., 2023), the original allocation of items was retained and the six items were scored as two three-item subscales. Functional presenteeism was assessed using the items: “Despite the period of protracted conflict, I was able to finish my difficult work tasks,” “Despite the period of protracted conflict, I was able to stay focused on achieving my goals at work,” and “Despite the period of protracted conflict, I felt energetic enough to complete my work” (Cronbach’s α = 0.86). Dysfunctional presenteeism was assessed using the items: “Due to the period of protracted conflict, I was distracted from enjoying my work,” “Due to the period of protracted conflict, the pressures of my job were much harder to manage,” and “Due to the period of protracted conflict, I felt helpless about finishing certain work tasks” (Cronbach’s α = 0.86).

Before administration, the adapted items underwent expert content evaluation to assess their clarity, relevance, and contextual appropriateness, consistent with recommendations for scale development (DeVellis and Thorpe, 2021). A panel of 19 healthcare professionals, including 11 nurses and 8 dietitians, all of whom held managerial positions and had extensive clinical experience during the protracted conflict, independently rated each item on these three criteria. Ratings were provided on a 5-point scale ranging from 1 (not at all clear/relevant/appropriate) to 5 (extremely clear/relevant/appropriate). Ratings of 4 or 5 were treated as agreement and dichotomized for the calculation of the Item Content Validity Index (I-CVI) and the Scale Content Validity Index using the average approach (S-CVI/Ave). In accordance with established recommendations, I-CVI values of 0.78 or higher were considered acceptable, while S-CVI/Ave values of 0.80 or higher indicated acceptable scale-level content validity and values of 0.90 or higher indicated excellent content validity (Polit et al., 2007; Polit and Beck, 2006). The adapted items demonstrated S-CVI/Ave values of 0.89 for clarity, 0.97 for relevance, and 0.96 for appropriateness. These findings support the content relevance and internal consistency of the adapted items but do not constitute evidence of full construct validation of the modified measure.

Perceived stress was measured using the National Aeronautics and Space Administration Task Load Index (NASA-TLX; Hart, 2006), assessing the subjective workload stemming from multi-source demands, including mental, physical, temporal, frustration, effort, and performance stressors. Each demand was assessed on a Likert-type scale ranging from 1 (very low workload) to 20 (very high workload), with higher scores indicating greater perceived demands on the respective dimension. An example item was “How fast and stressful has the last week been?”

Personal resilience was measured with the 6-item Brief Resilience Scale (BRS) (Smith et al., 2008). Participants rated their agreement with six items on a 5-point Likert-type scale ranging from 1 (totally disagree) to 5 (totally agree). An example item was “I am not easily discouraged by failures” (Cronbach’s α = 0.81).

Organizational trust was measured with the Organizational Trust 16-item scale (Tzafrir and Dolan, 2004). Participants were asked to rate on a Likert-type scale ranging from 1 (not at all) to 7 (to a very high extent) the extent to which they trust the organization. An example item was “My workplace management could be trusted in helping me solve problems that occurred during the period of war” (Cronbach’s α = 0.92).

Strain symptoms were assessed with the Shona Symptom Questionnaire (SSQ)-14 questionnaire (Patel et al., 1997). Items were rated on a 5-point Likert-type ranging from 1 (not at all) to 5 (always). An example item was “I had stomach aches” (Cronbach’s α = 0.89).

Control variables. Gender and years of professional tenure were considered as potential control variables based on previous evidence linking these characteristics to presenteeism (McGregor and Caputi, 2022). However, because the sample was predominantly female (86%), gender was not included as a control variable.

### Ethical considerations

The research protocol was approved by the institution’s ethics committee [Tel-Hai Academic college ethics committee number 19-6\2023]. Participants accessed the study through an anonymous online link. The first page of the survey included a participant information sheet and a detailed explanation of the study’s purpose, voluntary nature, and confidentiality assurances. Proceeding to the next page was considered informed consent. Participants were explicitly informed that they could withdraw from the study at any time without consequence and that all responses would remain anonymous and confidential.

### Data analysis

Data were analyzed using IBM SPSS Statistics version 27 for Windows to compute descriptive statistics (frequencies, means, and standard deviations) and Pearson correlations for preliminary associations. Path analysis was carried out using AMOS version 23 to test the hypothesized mediation in three consecutive stages. First, a full mediation model was tested in which perceived stress, personal resilience, and organizational trust were specified as interrelated explanatory variables, strain symptoms were modeled as a mediator, and all direct paths from the explanatory variables to the outcomes were constrained to zero (Figure 1). Second, following Bowen and Guo (2011) methodological recommendation to compare competing structural models, we tested a partial mediation model that included additional direct paths from personal resilience and organizational trust to functional presenteeism, and from perceived stress to dysfunctional presenteeism (Figure 2). Model comparisons between the full and partial mediation structures were conducted using chi-square difference tests and degrees of freedom to determine whether the more complex model provided a superior fit and theoretical robustness. Finally, bootstrap resampling (5,000 iterations) with bias-corrected 95% confidence intervals for standardized estimates was applied to assess the significance of direct, indirect, and total effects of the superior model from the second step.

**FIGURE 2:**
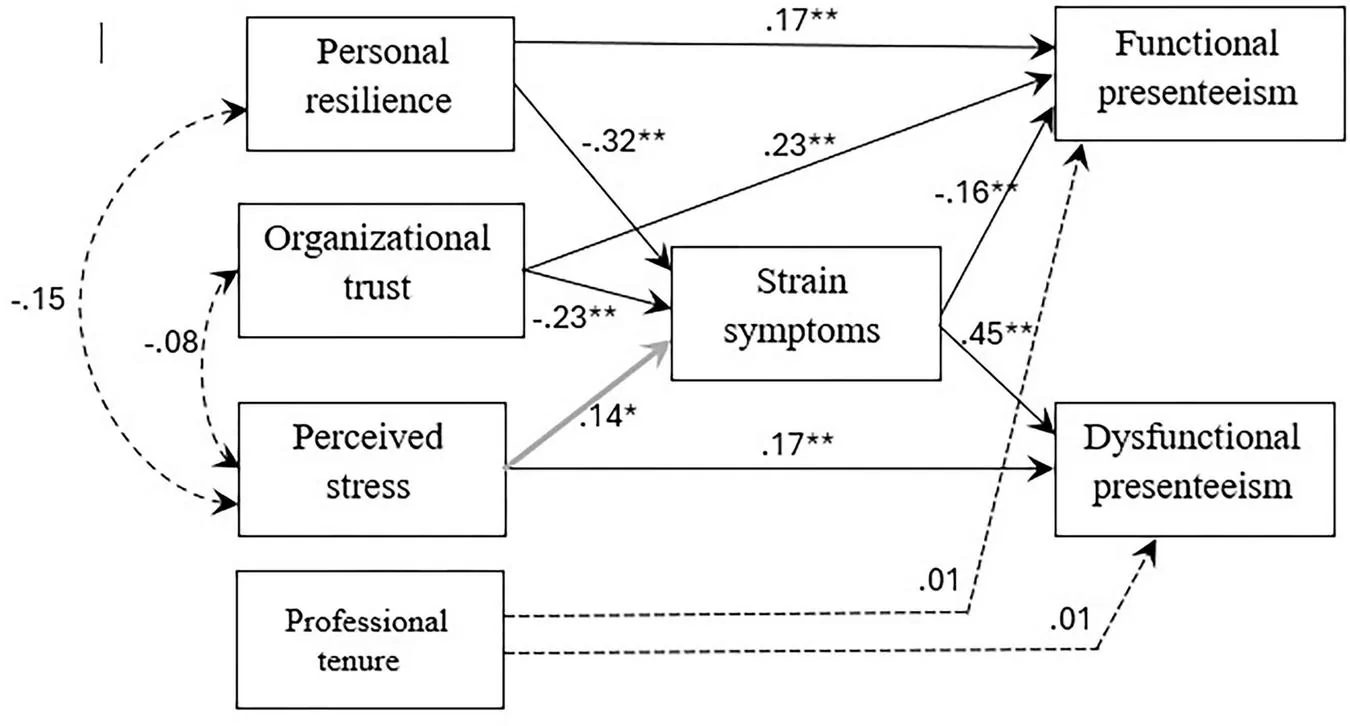
Alternative path model of the relationship between perceived stress, personal resilience, organizational trust and both forms of presenteeism as mediated by strain symptoms. Fit indices χ^2^(9) = 12.48, *p* = 0.188, CFI = 0.978, TLI = 0.949, RMSEA = 0.042. *P < 0.05; **P < 0.001. Standardized path coefficients are based on maximum likelihood (ML) estimates statistical inference regarding the significance of the direct and indirect effects is based on the bias-corrected bootstrap estimates reported in Supplementary Table 2. Solid black arrows denote paths that remained significant in both ML bootstrap analyze. Gray arrows denote paths that were significant in ML estimation but not supported by bias-corrected bootstrap confidence intervals. Dashed arrows denote paths from the control variable (professional tenure), which were retained in the model regardless of statistical significance.

There were no missing data in the explanatory, mediator, or outcome variables. Model parameters were estimated using the maximum likelihood (ML) method. Model fit was evaluated using the chi-square test, the Comparative Fit Index (CFI), the Tucker–Lewis Index (TLI), and the Root Mean Square Error of Approximation (RMSEA). CFI and TLI values of 0.90 or higher and RMSEA values of 0.08 or lower were considered indicative of acceptable model fit, whereas CFI and TLI values of 0.95 or higher and RMSEA values of 0.06 or lower were interpreted as indicating good-to-excellent fit. A non-significant chi-square test was also considered supportive of model fit (Hu and Bentler, 1999; Kline, 2023). Statistical significance was set at *p* < 0.05.

As a sensitivity analysis, the structural model was re-estimated with professional group (nurse vs. dietitian) included as an additional covariate to account for potential systematic differences between the professional groups and to examine whether professional affiliation materially affected the pattern of associations observed in the pooled sample.

## Results

### Sample characteristics

The sample consisted of 227 healthcare professionals, including 108 dietitians and 119 nurses. Most participants were married (67%) and female (86%) and had ages ranging from 24 to 69 years (*M* = 44, SD = 12). In terms of education, 54% held a bachelor’s degree, 35% held a master’s degree, and 10% held a doctoral degree. Participants’ tenure in the profession ranged from 1 to 45 years (*M* = 14.15, SD = 12.9).

### Descriptive findings

On the 1–5 response scale, participants reported moderately high functional presenteeism (*M* = 3.85, SD = 0.77) and a moderate level of dysfunctional presenteeism (*M* = 2.79, SD = 0.98). They also reported a moderate level of strain symptoms (*M* = 2.37, SD = 0.66) and a moderate level of personal resilience (*M* = 3.18, SD = 0.43) (Table 1). Table 1 also presents the correlations between the study variables. Functional presenteeism was negatively and significantly associated with strain symptoms (*r* = −0.29, *p* < 0.01) and positively and significantly associated with both personal resilience (*r* = 0.26, *p* < 0.01) and organizational trust (*r* = 0.30, *p* < 0.01). However, it was not significantly associated with perceived stress. In contrast, dysfunctional presenteeism was positively and significantly associated with strain symptoms (*r* = 0.49, *p* < 0.01) and perceived stress (*r* = 0.27, *p* < 0.01), and negatively and significantly associated with personal resilience (*r* = −0.27, *p* < 0.01) and organizational trust (*r* = −0.16, *p* < 0.05).

### Path analysis

Overall, the model demonstrated acceptable-to- good fit to the observed data: χ^2^(9) = 12.48, *p* = 0.188, CFI = 0.978, TLI = 0.949, and RMSEA = 0.042. Model adequacy was also supported by the Bollen–Stine bootstrap test, *p* = 0.227.

Figure 2 presents the standardized ML path coefficients for the proposed partial mediation model. Based on the ML estimates, perceived stress was significantly and positively associated with higher strain symptoms (β = 0.14, *p* = 0.02), whereas personal resilience (β = −0.32, *p* = 0.001) and organizational trust (β = −0.23, *p* = 0.001) were negatively associated with strain symptoms. In turn, strain symptoms were positively associated with dysfunctional presenteeism (β = 0.45, *p* < 0.001); and negatively associated with functional presenteeism (β = −0.16, *p* < 0.001).

To evaluate mediation, the proposed partial mediation model was first compared with a full mediation model in which the direct paths from the explanatory variables to the presenteeism outcomes were constrained to zero. The full mediation model demonstrated poor fit, χ^2^(12) = 37.45, *p* < 0.001, RMSEA = 0.131. A chi-square difference test confirmed that the partial mediation model provided a significantly better fit to the data [Δχ^2^(3) = 24.97, *p* < 0.001].

Indirect effects were then evaluated using 5,000 bootstrap resamples. Supplementary Table 2 presents bootstrap estimates, including 95% confidence intervals and *p*-values, which were used for statistical inference regarding the model paths and indirect effects.

The indirect association between perceived stress and dysfunctional presenteeism through strain symptoms was not statistically significant (β = 0.06, 95% CI [−0.004, 0.154], *p* = 0.058); therefore, Hypothesis 1 was not supported. Nevertheless, the direct association between perceived stress and dysfunctional presenteeism remained statistically significant (β = 0.17, 95% CI [0.048, 0.299], *p* = 0.005), indicating that this association was not mediated through strain symptoms.

By comparison, personal resilience demonstrated both a significant direct association with functional presenteeism (β = 0.17, 95% CI [0.043, 0.325], *p* = 0.004) and a significant positive indirect association through lower strain symptoms (β = 0.05, 95% CI [0.002, 0.123], *p* = 0.002), supporting Hypothesis 2. Similarly, organizational trust demonstrated both a significant direct association with functional presenteeism (β = 0.23, 95% CI [0.081, 0.339], *p* = 0.009) and a significant positive indirect association through lower strain symptoms (β = 0.04, 95% CI [0.007, 0.094], *p* = 0.002), supporting Hypothesis 3. Overall, these findings indicate that strain symptoms partially mediated the associations of personal resilience and organizational trust with functional presenteeism, whereas no significant mediation was observed for the association between perceived stress and dysfunctional presenteeism. The sensitivity model including professional group showed poorer fit than the primary model [χ^2^(14) = 38.24, *p* < 0.001, CFI = 0.874, TLI = 0.747, RMSEA = 0.088], while inclusion of professional group did not materially alter the substantive pattern of the hypothesized structural associations. The model was therefore not retained as the primary model. Full direct and indirect effects are presented in Supplementary Figure 1.

## Discussion

Grounded in Conservation of Resources (COR) theory (Hobfoll, 1989), this study examined factors associated with adaptive and maladaptive work functioning among healthcare professionals during protracted conflict, thereby contributing to the literature in several important ways. First, a key contribution lies in providing empirical support for the distinction between functional and dysfunctional presenteeism in healthcare within the context of protracted conflict. Echoing recent calls for a multidimensional conceptualization of presenteeism (Bezzina et al., 2023; Patel et al., 2023), the findings extend the traditional framing as solely harmful (Lohaus et al., 2021) and support a more nuanced view. In the present study, this distinction was examined using a contextual adaptation of the functional–dysfunctional presenteeism framework to capture continued work under the chronic psychological and situational strain of prolonged armed conflict. While prior studies have emphasized the risks of presenteeism resulting from overwork and internalized professional obligations (Baldonedo-Mosteiro et al., 2020), our results align with emerging evidence that presenteeism can also reflect adaptive coping and commitment to professional identity in the face of adversity (Mansour, 2024).

Second, the path analysis revealed two distinct relational patterns underlying functional and dysfunctional presenteeism. Regarding functional presenteeism, personal resilience and organizational trust each demonstrated significant direct associations and significant indirect associations through lower strain symptoms, indicating partial mediation. Notably, the direct associations were larger than the corresponding indirect associations, suggesting that lower strain symptoms explained only part of the relationships between these resources and functional presenteeism. The indirect effects are consistent with COR theory, which suggests that personal and organizational resources help preserve functioning by reducing resource depletion and strain (Hobfoll, 1989). At the same time, stronger direct effects indicate that resilience and organizational trust may support functional presenteeism through additional mechanisms not captured by strain symptoms. Consistent with Ungar (2011) concept of navigation, resilient employees may be better able to identify and access available organizational resources, while organizational trust may increase the perceived availability and reliability of such support.

Regarding dysfunctional presenteeism, the hypothesized indirect association of perceived stress through strain symptoms was not supported. Although perceived stress was significantly associated with strain symptoms in the maximum-likelihood estimates, the bootstrapped indirect effect was not statistically significant. Perceived stress nevertheless demonstrated a significant direct positive association with dysfunctional presenteeism, indicating that its relationship with impaired work functioning was not explained by strain symptoms in the present model. This direct association may reflect more immediate cognitive and behavioral consequences of perceived stress, such as difficulty concentrating, managing work demands, or sustaining task performance, which were not captured by the strain-symptom measure. In addition, particularly during protracted conflict, perceived stress may be linked to dysfunctional presenteeism through normative or cultural mechanisms. In healthcare organizations where norms such as “presence at all costs to avoid burdening colleagues” (Mohammadi et al., 2021) or beliefs that “a good professional never breaks” (Drach-Zahavy and Somech, 2010) prevail, employees may continue working despite impaired functioning (Lui et al., 2018). Thus, the findings suggest that perceived stress is associated with dysfunctional presenteeism primarily through direct or unmeasured pathways rather than through the hypothesized strain mechanism.

Third, the findings extend the application of the functional–dysfunctional presenteeism framework to the underexplored context of protracted emergencies, including prolonged conflict. While the COVID-19 pandemic highlighted the role of crisis-induced presenteeism (White-Means et al., 2022), empirical work during other prolonged emergencies remains scarce. The present findings suggest that this framework may also be useful for understanding work functioning under chronic stressors such as moral distress, safety threats, and continuous exposure to trauma, beyond the illness- and organizational pressure-related contexts traditionally emphasized in the presenteeism literature (Lui et al., 2018). By situating presenteeism within the ecological realities of ongoing conflict, the study offers a more contextually nuanced understanding of how healthcare professionals navigate competing demands, resource threats, and professional commitment under extreme conditions.

### Practical implications

The findings may inform healthcare organizations seeking to support staff well-being and work functioning during protracted conflict. The distinct patterns associated with dysfunctional and functional presenteeism suggest that organizational responses should address conditions linked to impaired work functioning while also strengthening personal and organizational resources associated with maintained functioning.

Dysfunctional presenteeism was directly associated with perceived stress and may reflect both resource depletion and normative pressures to remain at work despite impaired functioning. Because opportunities for real-time organizational adjustment may be limited during protracted conflict, advance preparedness may help reduce these pressures. Organizations may establish clear staffing and deployment protocols, cross-train personnel, and define thresholds for rest and rotation to limit excessive workload concentration during crises. Cultivating a culture that normalizes help-seeking and acknowledges the limits of endurance may also be important. Transparent communication regarding professional expectations, ethical boundaries, and the legitimacy of temporary withdrawal from duty may help challenge beliefs that “a good professional never breaks” (Drach-Zahavy and Somech, 2010) or that employees must remain present at all costs (Lui et al., 2018; Mohammadi et al., 2021).

Personal resilience and organizational trust were associated with functional presenteeism both directly and indirectly through lower strain symptoms, indicating that strain reduction represents one, but not the only, potential pathway supporting maintained work functioning. Healthcare organizations may therefore consider combining monitoring and early support for strain symptoms with broader efforts to strengthen organizational trust and employees’ coping resources. Routine attention to strain symptoms during supervision or shift handovers may facilitate early identification of support needs (White-Means et al., 2022). The Anticipate–Plan–Deter framework and its PsySTART self-triage tool may offer one approach for identifying acute distress and team-level risks (Schreiber et al., 2019). Open communication, psychological safety, and consistent managerial support may strengthen organizational trust (Drach-Zahavy et al., 2022; Gillespie et al., 2020), while peer support, mindfulness, and stress-management initiatives may help support coping during crises (Curtin et al., 2022; Debie et al., 2024).

### Limitations and future research

While this study advances understanding of functional and dysfunctional presenteeism during a period of protracted conflict, several limitations should be acknowledged. First, the cross-sectional design, based on a single time point of data collection, precludes causal inference. Although mediation paths were modeled and the proposed pathways were theory-driven and grounded in COR theory, temporal sequencing cannot be confirmed, and the dynamics among strain, resilience, and trust remain unobserved.

Second, all study variables were assessed using self-report questionnaires, which increases the possibility of common method variance and may have inflated the observed associations among the study variables (Podsakoff et al., 2003), Although perceptual constructs such as strain are appropriately assessed using self-report measures, the exclusive reliance on a single data source limits the ability to rule out shared method effects.

Third, participants were recruited using convenience and snowball sampling. Although these approaches were appropriate under wartime conditions, they may have introduced selection bias and may limit the generalizability of the findings. However, these recruitment strategies also enabled access to healthcare professionals across diverse clinical settings during an ongoing conflict, reaching participants who would have been difficult to recruit through a single organization or healthcare system.

Fourth, the sample included only nurses and dietitians and was predominantly female. Although both professions maintained direct patient-care responsibilities during the protracted conflict, their roles, work environments, and exposure to conflict-related demands may have differed. The sensitivity analysis adjusting for professional group did not materially alter the substantive pattern of associations; however, the study was not designed to test whether specific structural pathways differed between professions. Conversely, including two healthcare professions rather than a single occupational group increased the heterogeneity of the sample and allowed the proposed theoretical model to be examined across frontline clinical professionals rather than within a single discipline. Accordingly, caution is warranted when generalizing the findings to other healthcare professions or to more gender-balanced populations.

Finally, because the SPS-6 was adapted to the context of protracted armed conflict, its dimensional structure and construct validity were not independently established through confirmatory factor analysis. Although expert evaluation supported the clarity, relevance, and contextual appropriateness of the adapted items, and both subscales demonstrated high internal consistency, future research should undertake a full psychometric evaluation of the adapted measure. Accordingly, findings based on the adapted SPS-6 should be interpreted as reflecting context-specific work functioning rather than as directly equivalent to classical illness-related presenteeism.

Future longitudinal or mixed-methods research is needed to validate the temporal processes implied by the mediation model and to explore how functional and dysfunctional presenteeism evolve over time. Extending the sample to additional professional groups will further strengthen generalizability.

## Conclusion

This study extends the functional–dysfunctional presenteeism framework to the context of protracted conflict using a contextually adapted assessment of work functioning and provides partial support for a resilience-oriented model among healthcare professionals. Grounded in Conservation of Resources (COR) theory, the findings indicate distinct patterns of association: perceived stress was directly associated with dysfunctional presenteeism, whereas personal resilience and organizational trust were associated with functional presenteeism through lower strain symptoms. These findings highlight the importance of both individual and organizational resources in understanding how healthcare professionals maintain work functioning under prolonged adversity.

## Data Availability

The raw data supporting the conclusions of this article will be made available by the authors, without undue reservation.
