# Supplementary material for: Contextually adapted functional and dysfunctional presenteeism during protracted conflict
Source: Front Psychol. 2026 Sep 16;17:1903610. doi: 10.3389/fpsyg.2026.1903610 (PMC13623933; doi:10.3389/fpsyg.2026.1903610)
Supplement: Supplementary file 1 [file Table_1.docx]

Supplementary Table S2. Bias-corrected bootstrap estimates of the standardized direct, indirect, and total effects of the structural model

| Effect | Estimate β | 95% CI | p-value |
| --- | --- | --- | --- |
| Indirect effects |  |  |  |
| Personal resilience → Strain symptoms → Functional presenteeism | .05 | [.002, .123] | .002 |
| Organizational trust → Strain symptoms → Functional presenteeism | .04 | [.007, .094] | .002 |
| Perceived stress → Strain symptoms → Dysfunctional presenteeism | .06 | [-.004, .154] | .058 |
| Direct effects |  |  |  |
| Personal resilience → Strain symptoms | -.32 | [-.424, -.193] | .003 |
| Organizational trust → Strain symptoms | -.23 | [-.363, -.095] | .002 |
| Perceived stress → Strain symptoms | .14 | [-.016, .312] | .070 |
| Personal resilience → Functional presenteeism | .17 | [.043, .325] | .004 |
| Organizational trust → Functional presenteeism | .23 | [.081, .339] | .009 |
| Perceived stress → Dysfunctional presenteeism | .17 | [.048, .299] | .005 |
| Strain symptoms → Functional presenteeism | -.17 | [-.335, -.002] | .048 |
| Strain symptoms → Dysfunctional presenteeism | .45 | [.323, .573] | .003 |
| Professional tenure → Functional presenteeism | .01 | [-.107, .128] | .839 |
| Professional tenure → Dysfunctional presenteeism | .01 | [-.097, .129] | .822 |
| Total effects |  |  |  |
| Personal resilience → Functional presenteeism | .22 | [.089, .350] | .002 |
| Organizational trust → Functional presenteeism | .27 | [.133, .396] | .003 |
| Perceived stress → Dysfunctional presenteeism | .23 | [.082, .364] | .001 |

Note. Standardized coefficients, 95% bias-corrected bootstrap confidence intervals, and p-values are presented. Bootstrap estimates were used to evaluate the significance of the direct and indirect effects.
